# The effect of high-intensity interval training and moderate-intensity continuous training on cardiorespiratory function in healthy elderly individuals: Systematic review and meta-analysis

**DOI:** 10.1097/MD.0000000000047101

**Published:** 2026-01-09

**Authors:** Rui Chu, Mingming Li, Caiwei Zhu, Yinuo Du, Shouzhi Wu

**Affiliations:** a Anhui Polytechnic University, Wuhu, Anhui, China; b Hefei Preschool Education College, Hefei, Anhui, China; c Wannan Medical College, Wuhu, Anhui, China.

**Keywords:** aerobic exercise, cardiopulmonary function, continuous training, high-intensity interval training, public health

## Abstract

**Background:**

This study aims to systematically evaluate the intervention effects of high-intensity interval training (HIIT) and moderate-intensity continuous training (MICT) on cardiovascular and pulmonary functions in healthy elderly individuals, providing evidence-based recommendations for the development of exercise prescriptions for this population.

**Methods:**

A systematic search was conducted in the PubMed, Web of Science, Cochrane Library, and Google Scholar databases (through March 2025) to identify randomized controlled trials that investigated the effects of HIIT and MICT on cardiovascular and pulmonary functions in elderly individuals. Data were analyzed using RevMan 5.4 and Stata 15.1, employing a random-effects model to calculate the pooled effect size (weighted mean difference) and its 95% confidence interval (95% CI) for maximal oxygen uptake.

**Results:**

A total of 16 studies (n = 1434) were included. In the MICT group (12 studies), maximal oxygen uptake levels were significantly improved (mean difference [MD] = 1.22, 95% CI: 0.90–1.53). In the HIIT group (11 studies), the improvement was more pronounced (MD = 1.62, 95% CI: 1.10–2.13). In studies that included both HIIT and MICT (7 studies), HIIT demonstrated significantly superior improvements compared to MICT (MD = 1.17, 95% CI: 0.52–1.82). Subgroup analysis revealed that the optimal MICT protocol consisted of a moderate duration (>3 months and <6 months), 3 sessions per week, and a session duration of ≥60 minutes. The optimal HIIT protocol involved a moderate duration (>3 months and <6 months), 4 sessions per week, and a session duration of 21 to 39 minutes.

**Conclusion:**

Both HIIT and MICT effectively improve cardiovascular and pulmonary function in elderly individuals, with HIIT yielding more favorable results. MICT is suitable for long-term training in individuals with good tolerance, while HIIT is better suited for short-term interventions for those able to tolerate high-intensity exercise. In clinical practice, the appropriate training regimen should be selected based on individual health status.

## 1. Introduction

Cardiorespiratory fitness is a key physiological indicator used to assess the efficiency of oxygen transport and utilization in the body. It is determined by factors such as cardiac output, pulmonary ventilation capacity, and the ability of peripheral tissues to extract oxygen. The level of cardiorespiratory fitness directly impacts physical activity levels and the risk of chronic diseases.^[1]^ Common clinical measures of cardiorespiratory fitness include the 6-minute walk distance and forced vital capacity, among others. Among these, maximal oxygen uptake (VO_2max_) is considered the gold standard for assessing cardiorespiratory endurance. This indicator reflects the body’s ability to take up, transport, and utilize oxygen per unit of time.^[2]^ Furthermore, numerous epidemiological studies have confirmed that cardiorespiratory fitness, as an objective measure, is positively correlated with all-cause mortality, the incidence of cardiovascular diseases, diabetes, and other chronic conditions in various populations.^[3–5]^

It is well-established that after the age of 30, due to the natural decline in physiological function and reduced physical activity, VO_2max_ decreases by 5% to 15% every decade. By the age of 70, this decline can reach up to 50%.^[6–9]^ As a result, addressing the decline in the health of elderly individuals and developing targeted exercise interventions to improve their cardiorespiratory fitness has become a critical issue that requires urgent attention.

In recent years, high-intensity interval training (HIIT) and moderate-intensity continuous training (MICT) have emerged as mainstream exercise modalities for improving cardiorespiratory fitness, with their health benefits widely studied. HIIT refers to a training method that involves short bouts of exercise at high intensities (≥85% VO_2max_, or ≥90% HR_max_), interspersed with low-intensity exercises or complete rest between high-intensity intervals.^[10]^ MICT, on the other hand, involves continuous, longer-duration exercise at moderate intensities (60–75% VO_2max_ or 60–70% HR_max_) without breaks.^[11]^ Several studies have shown that both HIIT and MICT significantly improve cardiorespiratory fitness (measured primarily by VO_2max_) in adolescents, adults, and patients with chronic diseases such as cardiovascular disease and metabolic syndrome.^[12–16]^

However, research specifically targeting healthy elderly populations remains limited. First, many existing studies have small sample sizes (n < 100), leading to insufficient statistical power. Second, there is considerable variation in the intervention parameters used across studies (such as training duration, frequency, and intensity), which reduces the comparability of results.^[17,18]^ Moreover, there is no consensus on the comparative effectiveness of HIIT and MICT in improving cardiorespiratory fitness. Some studies suggest that HIIT is more effective than MICT,^[19,20]^ while other studies report the opposite,^[21]^ and yet others find no significant difference between the 2.^[22,23]^ Additionally, most of these studies focus on younger populations or athletes, with limited research on healthy elderly individuals. Although meta-analyses have synthesized the effects of HIIT and MICT in non-elderly populations,^[24–26]^comprehensive analyses specifically focusing on healthy elderly individuals are still scarce, particularly regarding the quantification of dose-response relationships (such as optimal intervention duration, frequency, and intensity).

Based on this, the present study employs a meta-analysis approach to conduct a comprehensive and systematic quantitative review of existing studies. The goal is to synthesize and quantify the overall effect of HIIT and MICT on improving Cardiorespiratory fitness in healthy elderly individuals, analyze the dose–response relationship between exercise intervention parameters and the overall effect size, and compare the effectiveness of HIIT and MICT in enhancing cardiorespiratory fitness in this population. This analysis aims to provide practical evidence and theoretical references for the development of health intervention strategies and the implementation of exercise practices for elderly individuals.

## 2. Methods

In accordance with the Preferred Reporting Items for Systematic Reviews and Meta-Analyses (PRISMA) guidelines for systematic reviews and meta-analyses,^[27]^ the protocol was registered at the University of York, Centre for Reviews and Dissemination PROSPERO database: Registration No. CRD420251037775 (https://www.crd.york.ac.uk/PROSPERO/view/CRD420251037775). This study is a systematic review and meta-analysis of existing randomized controlled trials (RCTs) and does not involve direct human or animal experimentation. Since the research synthesizes previously published data, it does not require additional ethical approval from an institutional review board or ethics committee. All included trials were expected to have obtained original ethical clearance during their respective study conduct.

### 2.1. Search strategy

This study combined keywords such as “Aged,” “High-Intensity Interval Training,” and “Cardiorespiratory Fitness” with free terms like “elderly,” “Exercises,” and “Fitness, Cardiorespiratory” to form the search query. Two independent researchers (MML and YND, the authors of the 2 articles received relevant training before work) conducted the literature search in a double-blind manner, using electronic databases such as PubMed, Web of Science, Cochrane Library, and Google Scholar. The search was limited to RCTs that investigated the effects of HIIT or MICT on the cardiorespiratory function of healthy elderly individuals. The search cutoff date was set to February 15, 2025.

After completing the literature search and collection, the titles and abstracts of all identified articles were evaluated according to predefined inclusion and exclusion criteria. Duplicate or clearly irrelevant studies were removed from the database list. Full-text articles were then retrieved, and 2 researchers independently assessed the quality of the studies and extracted the relevant data. In case of any disagreements, a third researcher was consulted, and a discussion group was formed to resolve the issues.

### 2.2. Inclusion and exclusion criteria

Inclusion criteria:

1. Participants: elderly individuals aged ≥60 years who are in good health and capable of engaging in a certain amount of physical activity.
2. Interventions: participants must undergo at least 2 weeks of HIIT or MICT. HIIT is generally defined as training involving exercise intensities near “maximum effort,” such as ≥90% VO_2max_ or 85% to 95% HR_max_, with short bursts of high-intensity exercise alternated with periods of active recovery.^[28]^ MICT typically refers to continuous training at moderate intensity (55–69% HR_max_ or 40–59% VO_2max_) without recovery breaks for an extended period.^[29]^
3. Outcome measures: at least 1 indicator of cardiorespiratory function (such as VO_2max_ or 6-minute walk distance) must be included.
4. Language: articles must be written in English.
5. Study design: the study must be a RCT or a controlled clinical trial.

Exclusion criteria:

1. Mixed interventions: studies that combine other interventions (e.g., strength resistance training, pharmacological treatment).
2. Inappropriate participants: animal studies, or studies where participants are adults, children, or elderly individuals with a history of disease.
3. Incomplete or inaccessible data: studies with missing or unretrieveable data.
4. Publication type: unpublished studies or gray literature (including conference proceedings, theses, patents, etc).

### 2.3. Data extraction

Two researchers (MML and YND) independently extracted relevant data from the included studies using a double-blind approach. The extracted data included the following information: the 1st author, publication year, participant characteristics (sample size, age, physical condition), intervention protocol (including frequency and duration), and study outcomes. For the interventions targeting cardiorespiratory health, only the final values of outcome measures were compared with baseline values. Data were extracted in the form of the mean and standard deviation before and after the intervention.

### 2.4. Quality assessment

Two researchers (MML and YND) independently assessed the quality and risk of bias of the 16 included studies using an adapted 8-item risk checklist based on the PRISMA standards.^[30]^ Each item in the checklist had 3 response options: “clearly described” (1 point), “none,” and “unclear or insufficient description” (0 points). The 8 standards used for quality assessment and bias risk evaluation were as follows:

1. Were the sample inclusion criteria clearly defined?
2. Were participants randomly assigned to groups?
3. At baseline, were there no significant differences between groups in terms of key outcome measures?
4. Were all assessors of primary outcome measures blinded?
5. Was targeted statistical analysis used for the primary outcome measure data?
6. Was attrition of participants reported, with a dropout rate of <20%?
7. Was a sample size calculation performed, and did the study have sufficient power to detect changes in the primary outcome measures?
8. Were summary results for each group, estimated effect sizes (group differences), and their precision (e.g., 95% confidence intervals) reported?

Studies that scored between 7 and 8 points were classified as low risk; studies with scores between 4 and 6 points were classified as moderate risk; and studies with scores below 4 points were classified as high risk.

### 2.5. Data analysis

The study used RevMan (version 5.4; The Cochrane Collaboration, London, UK) and Stata (version 15.1; StataCorp LLC, College Station) software to analyze the outcome measures of the included studies, aiming to determine the effects of HIIT and MICT on the cardiorespiratory health of healthy elderly individuals, as well as the dose–response relationship. Since the outcome measures in the included studies were all continuous variables with consistent measurement units, the weighted mean difference (WMD) and 95% confidence intervals (CI) were chosen as the effect size for the meta-analysis.

For heterogeneity assessment, the *I*² statistic was used to measure the extent of heterogeneity across studies. When *I*² < 25% and the *Q* test indicated *P* < .05, low heterogeneity between studies was assumed. When *I*² was between 25% and 50%, with a *P*-value < .05 in the *Q* test, moderate heterogeneity was considered. If *I*^2^ ≥ 50% and *P* < .05, high heterogeneity was assumed.^[31]^

To assess publication bias, statistical tests such as Begg and Egger tests were conducted. If potential publication bias was detected, a trim-and-fill method was applied to assess the stability of the overall effect size.^[32]^ Additionally, sensitivity analysis was performed by sequentially excluding studies to evaluate the robustness of the meta-analysis results.

Subgroup analysis was also conducted to explore the differential effects of various interventions, including intervention type, frequency, duration, and cycle, on the cardiorespiratory health of elderly individuals.

## 3. Results

### 3.1. Literature search results

**Figure 1.**
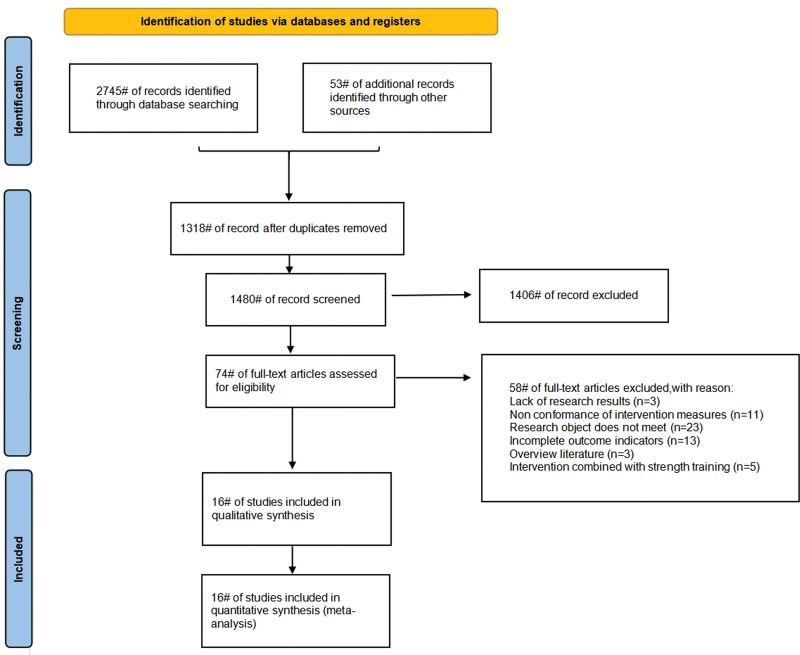
Literature selection process. The systematic literature screening process based on PRISMA process. An initial search yielded 2798 articles. After deduplication, title abstract screening, and full-text evaluation, 16 randomized controlled trials (n = 1434) were ultimately included. Provide a detailed presentation of the reasons for exclusion and the number of literature in each stage. PRISMA = Preferred Reporting Items for Systematic Reviews and Meta-Analyses.

A comprehensive search was conducted across multiple electronic databases, initially yielding 2798 relevant studies. After removing duplicates, 1318 studies were excluded, leaving 1480 studies for further review. Titles and abstracts were then screened, resulting in the exclusion of 1406 studies that were deemed irrelevant. Finally, full-text assessments were performed based on the predefined inclusion and exclusion criteria, resulting in the inclusion of 16 studies for data extraction. The detailed selection process is shown in Figure 1.

### 3.2. Characteristics of the studies

Table 1 provides a detailed summary of the participant characteristics, experimental grouping, intervention measures, intervention frequency and duration, as well as the outcome measures used to assess changes in cardiorespiratory function in elderly individuals. A total of 16 studies, involving 1434 participants, were included in this meta-analysis. All participants were healthy elderly individuals, aged 60 to 80 years, who were sedentary and lacked regular physical exercise.^[33–48]^

**Table 1:** Literature inclusion characteristics.

Author/references	Subject characteristics	Age (yr)	Groups	Interventions	Duration (wk)	Frequency (d/wk)	CRF outcomes
Uusitalo et al^[33]^	Sedentary older adults (n = 117)	68 ± 5	MICT (n = 59) CG (n = 58)	MICT: skiing, swimming, and cycling for 45–60 min at 40–60% of VO_2max_	48	3	VO_2max_ (mL kg^-1^ min^-1^)
Simonsson et al^[34]^	Inactive older adults (n = 68)	72.5 ± 6.5	HIIT (n = 34) MICT (n = 34)	HIIT: 10 × 6 s of training with 95% HRR interspersed with 54 s active recovery; MICT: 3 × 8 min of training at 70% HRR intensity, interspersed with 3 min of active recovery.	12	2	VO_2max_ (mL kg^-1^ min^-1^)
Haynes et al^[35]^	Healthy older adults (n = 40)	62.7 ± 7	MICT (n = 20) CG (n = 20)	MICT: perform 50 min of underwater walking at an intensity of 55–65% HRR.	24	3	VO_2max_ (mL kg^-1^ min^-1^)
Sian et al^[36]^	Healthy older adults (n = 20)	75 ± 5	HIIT (n = 10) CG (n = 10)	HIIT: perform 5 × 1 min of body weight exercise with an intensity of 85% HR_max_, interspersed with 90 s of active recovery.	4	3	VO_2max_ (mL kg^-1^ min^-1^)
Letnes et al^[37]^	Inactive older adults (n = 398)	73 ± 2.1	HIIT (n = 210) MICT (n = 188)	HIIT: 4 × 4 min at 90% HR_max_ interspersed with 3 × 8 min active recovery at 70% of HR_max_; MICT: train continuously for 50 min at 70% HR_max_ intensity.	48	2	VO_2max_ (mL kg^-1^ min^-1^)
Hwang et al^[38]^	Healthy older adults (n = 34)	65 ± 1	HIIT (n = 17) MICT (n = 17)	HIIT: 4 × 4 min at 90% HR_max_ interspersed with 3 × 3 min active recovery at 70% of HR_max_; MICT: lsocaloric continuous exercise at 70% HR_max_ performed on a non-weight-bearing all-extremity ergometer.	8	4	VO_2max_ (mL kg^-1^ min^-1^)

Study	Subject characteristics	Age (yr)	Groups	Interventions	Duration (wk)	Frequency (d/wk)	CRF outcomes
Frost et al^[39]^	Cognitively noemal older adults (n = 68)	69.1 ± 5.2	MICT (n = 34) CG (n = 34)	MICT: cycling for 50 min with an aerobic capacity of 50–60%	24	2	VO_2max_ (mL kg^-1^ min^-1^)
Garcia et al^[40]^	Inactive older women (n = 36)	67.8 ± 6.2	HIIT (n = 18) MICT (n = 18)	HIIT: 60 min high-speed interval training with RPE intensity of 12–18 points; MICT:60 min of uniform and continuous training at RPE intensity of 6–14 points.	18	2	VO_2max_ (mL kg^-1^ min^-1^)
Fosstveit et al^[41]^	Healthy older adults (n = 233)	67 ± 6	HIIT (n = 122) CG (n = 111)	HIIT:38 min of interval training at an intensity >80% HR_peak_, including 10 × 2 min of training and 2 min rest between sets.	24	3	VO_2max_ (mL kg^-1^ min^-1^)
Rohmansyah et al^[42]^	Inactive older women (n = 24)	60 ± 5	HIIT (n = 12) MICT (n = 12)	HIIT:4 × 4 min of cycling exercise at 90–95% HR_max_, followed by 3 min of active recovery at 50–70% HR_max_; MICT: continuously cycling for 47 min at an HR_max_ intensity of 70–75%.	16	3	VO_2max_ (mL kg^-1^ min^-1^)
Brown et al^[43]^	Cognitively noemal older adults (n = 68)	68.4 ± 4.2	MICT (n = 34) CG (n = 34)	MICT: cycling for 50 min with 6 to 14 Borg scale of perceived exertion.	24	2	VO_2max_ (mL kg^-1^ min^-1^)

Study	Subject characteristics	Age (yr)	Groups	Interventions	Duration (wk)	Frequency (d/wk)	CRF outcomes
Guadagni et al^[44]^	Healthy older adults (n = 233)	65.9 ± 6.4	MICT (n = 103) CG (n = 130)	MICT: aerobic exercise at a VO_2max_ intensity of 60–70% for 40 min continuously.	24	3	VO_2max_ (mL kg^-1^ min^-1^)
Herrod et al^[45]^	Healthy older adults (n = 20)	69 ± 3	HIIT (n = 10) CG (n = 10)	HIIT: train on a stationary bicycle at a peak power output intensity of 90–110% for 5 × 1 min.	6	3	VO_2max_ (mL kg^-1^ min^-1^)
Klonizakis et al^[46]^	Postmenopausal women (n = 18)	70 ± 15	HIIT (n = 11) MICT (n = 7)	HIIT: 10 × 1 min cycling at 100% of peak power output interspersed with 1 min active recovery; MICT: 40 min of cycling at 65% of peak power output.	4	3	VO_2max_ (mL kg^-1^ min^-1^)
Kim et al^[47]^	Sedentary older adults (n = 35)	64 ± 1	HIIT (n = 17) MICT (n = 18)	HIIT: 4 × 4 min at 90% HR_max_ interspersed with 3 × 3 min active recovery at 70% of HR_max_; MICT: lsocaloric exercise at 70% HR_max_ performed on an all-extremity non-weight-bearing ergometer.	8	4	VO_2max_ (mL kg^-1^ min^-1^)
Herbert et al^[48]^	Sedentary older adults (n = 22)	62 ± 2	HIIT (n = 22)	HIIT: train on a stationary bicycle at a peak power output intensity of 80% −90% for 6 × 30 s, interspersed with a 3-min active recovery period.	6	2	VO_2max_ (mL kg^-1^ min^-1^)

CG = control group, CRF = cardiorespiratory fitness, HIIT = high-intensive interval training, HR_max_ = maximal heart rate, HR_peak_ = peak heart rate, HRR = heart rate reserve, MICT = moderate-intensity continuous training, PPO = peak power output, RPE = Borg rating of perceived exertion, VO_2max_ = maximal oxygen uptake.

Regarding the interventions, 12 studies employed MICT,^[33–35,37–40,42–44,46,47]^ with intervention durations primarily ranging from 40 to 60 minutes. The intensity of MICT was generally maintained at 40% to 70% of VO_2max_ or 55% to 75% of HR_max_. Eleven studies used HIIT,^[34,36–38,40–42,45–48]^ with exercise intensities of 80% to 95% HR_max_ or 80% to 110% peak power output for short bursts of high-intensity exercise, interspersed with periods of active recovery or complete rest during the training sessions.

All 16 studies used VO_2max_ (mL·kg^−1^·min^−1^) as the primary outcome measure to assess the effects of HIIT and MICT on the cardiorespiratory health of elderly individuals. The data for VO_2max_ were reported as means and SD. The intervention periods typically ranged from 6 to 24 weeks, with 4 studies having intervention durations of 4 weeks or 48 weeks. The overall intervention frequency ranged from 2 to 4 sessions per week.

### 3.3. Risk of bias

According to Table 2, the researchers performed a quality and risk of bias assessment of the 16 included studies using the 8-item risk checklist adapted from the PRISMA standards. The evaluation results indicated that 2 studies had a high risk of bias,^[42,45]^ ten studies had a moderate risk of bias,^[33,35–37,40,41,43,44,46,48]^ and 4 studies had a low risk of bias.^[34,38,39,47]^

**Table 2:** Quality evaluation and bias risk analysis of 16 studies.

Study	n	Age	1	2	3	4	5	6	7	8	Total	Risk of bias
Uusitalo et al^[33]^	117	68 ± 5	0	1	1	0	1	1	0	1	5	Medium risk
Simonsson et al^[34]^	68	72.5 ± 6.5	1	1	1	1	1	1	0	1	7	Low risk
Haynes et al^[35]^	40	62.7 ± 7	1	1	1	0	0	1	1	1	6	Medium risk
Sian et al^[36]^	20	75 ± 5	1	1	1	0	0	1	0	1	5	Medium risk
Letnes et al^[37]^	398	73 ± 2.1	1	1	1	0	1	0	0	1	5	Medium risk
Hwang et al^[38]^	34	65 ± 1	1	1	1	1	0	1	1	1	7	Low risk
Frost et al^[39]^	68	69.1 ± 5.2	1	1	0	1	1	1	1	1	7	Low risk
Garcia et al^[40]^	36	67.8 ± 6.2	1	1	1	0	1	0	0	1	5	Medium risk
Fosstveit et al^[41]^	233	67 ± 6	1	1	1	0	1	1	0	1	6	Medium risk
Rohmansyah et al^[42]^	24	60 ± 5	1	1	0	0	1	0	1	0	4	High risk
Brown et al^[43]^	68	68.4 ± 4.2	1	1	0	0	1	1	1	1	6	Medium risk
Guadagni et al^[44]^	233	65.9 ± 6.4	1	0	1	0	1	1	0	1	5	Medium risk
Herrod et al^[45]^	20	69 ± 3	0	0	1	0	1	1	0	1	4	High risk
Klonizakis et al^[46]^	18	70 ± 15	1	1	1	0	1	1	0	1	6	Medium risk
Kim et al^[47]^	35	64 ± 1	1	1	1	1	0	1	1	1	7	Low risk
Herbert et al^[48]^	22	62 ± 2	1	0	1	0	1	1	0	1	5	Medium risk

Criteria: (1) eligibility criteria were specified; (2) participants were randomly allocated to groups; (3) the groups were similar at baseline regarding the primary outcome(s); (4) there was blinding of all assessors who measured the primary outcome(s); (5) data for primary outcome(s) were analyzed by “intention to treat”; (6) dropout for primary outcome(s) was described, with <20% dropout of participants; (7) conducted the sample size calculations and the study was adequately powered to detect changes in the primary outcome(s); and (8) summary results for each group plus estimated effect size (difference between groups) and its precision (e.g., 95% CI) were reported.

### 3.4. Meta-analysis results

#### 3.4.1. Effect of MICT on cardiorespiratory function in healthy elderly individuals

**Figure 2.**
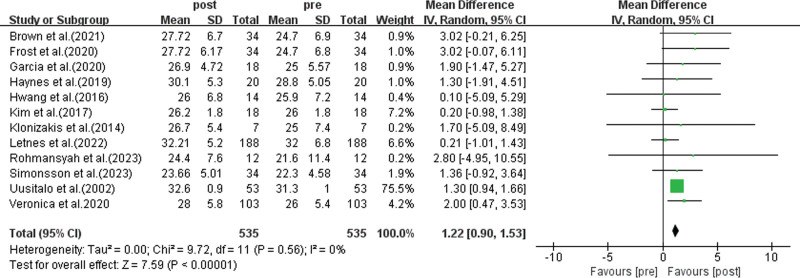
Analysis of the impact of MICT on the cardiorespiratory function of healthy elderly. Meta-analysis results of 12 studies (n = 535). MICT significantly improved the maximum oxygen uptake (VO_2max_) of healthy elderly individuals [WMD = 1.22, 95% CI: 0.90–1.53, *P* < .01], with no heterogeneity (*I*² = 0%). HIIT = high-intensity interval training, MICT = moderate-intensity continuous training, VO2max = maximal oxygen uptake, WMD = weighted mean difference.

Among the included studies, 12 studies reported the impact of MICT on the cardiorespiratory function of healthy elderly individuals, with a total of 535 participants.^[33–35,37–40,42–44,46,47]^ The meta-analysis results (as shown in Fig. 2) revealed a pooled effect size of: mean difference (MD) = 1.22, 95%: 0.90 to 1.53, *P* < .01, indicating statistically significant differences. This suggests that MICT can effectively improve the cardiorespiratory function of healthy elderly individuals.

Furthermore, the heterogeneity test showed *I*^2^ = 0%, *P* = .56, indicating no significant heterogeneity among the study results. To assess the potential for publication bias, Egger test and Begg test were performed on the included studies. The results of Egger test indicated *P* = .646 (*P* > .05), and the results of Begg test showed *P* = .954 (*P* > .05), suggesting that there was no publication bias, and the results are reliable.

#### 3.4.2. Effect of HIIT on cardiorespiratory function in healthy elderly individuals

**Figure 3.**
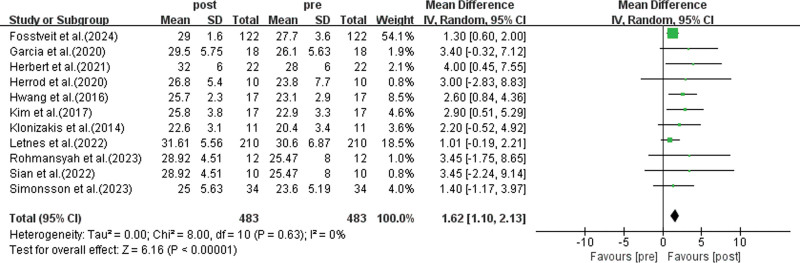
Analysis of the impact of HIIT on the cardiorespiratory function of healthy elderly. Meta-analysis results of 11 studies (n = 483). HIIT significantly increased VO_2max_ [WMD = 1.62, 95% CI: 1.10–2.13, *P* < .01], with no heterogeneity (*I*^2^ = 0%). HIIT = high-intensity interval training, VO2max = maximal oxygen uptake, WMD = weighted mean difference.

Among the included studies, 11 studies employed HIIT as the exercise intervention, involving a total of 483 participants.^[34,36–38,40–42,45–48]^ The meta-analysis results (as shown in Fig. 3) revealed a pooled effect size of: MD = 1.62, 95%: 1.10 to 2.13, *P* < .01, indicating statistically significant differences. This suggests that HIIT, as a short-duration, high-intensity training method, can effectively improve the cardiorespiratory function of healthy elderly individuals.

Furthermore, the heterogeneity test results showed *I*^2^ = 0%, *P* = .63, indicating no significant heterogeneity between the studies.

To assess whether publication bias exists in the included studies, Egger and Begg tests were performed. Although the Begg test showed *P* = .533 (*P* > .05), indicating no significant bias, the Egger test revealed *P* = .003 (*P* < .05), suggesting that there is some degree of publication bias among the included studies. Therefore, further analysis using the trim-and-fill method was conducted to test the stability of the study results.

**Figure 4.**
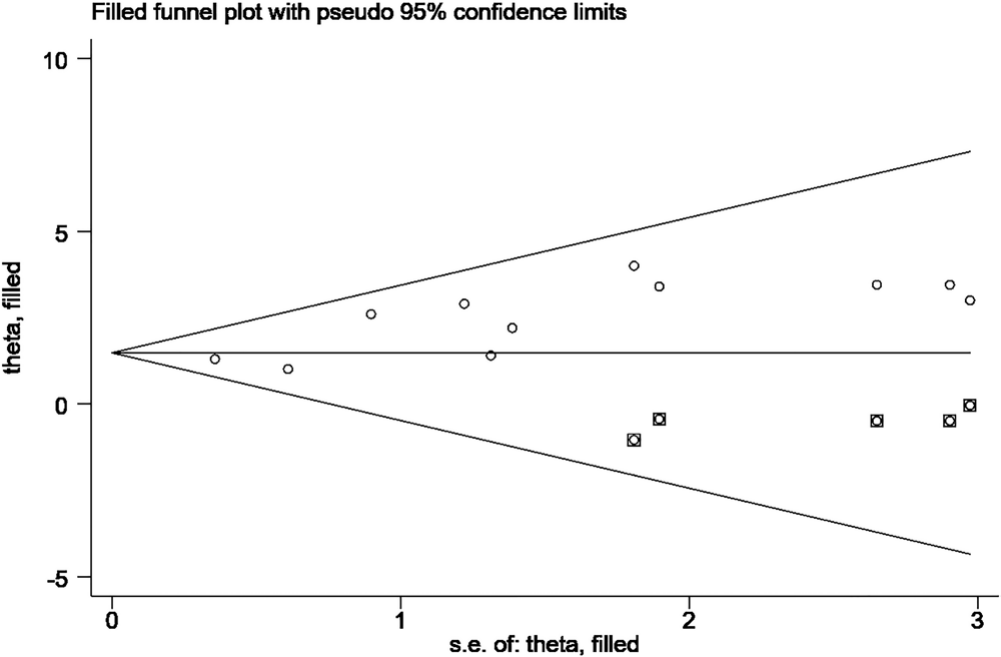
Application results of shear compensation method (including 11 included studies and 5 filled studies). Correction analysis after filling in 5 potential missing studies (hollow circles) using the pruning method. There was no significant difference in the effect size before and after correction (original WMD = 1.62 vs corrected WMD = 1.48, *P* = .640), confirming the stability of the conclusion that HIIT improves VO_2_max. HIIT = high-intensity interval training, VO_2max_ = maximal oxygen uptake, WMD = weighted mean difference.

After adding 5 potential missing studies using the trim-and-fill method (Fig. 4), the random-effects model analysis indicated no significant difference between the results before (MD = 1.62, 95% CI = 1.10–2.13) and after applying the trim-and-fill method (MD = 1.48, 95% CI = 0.981–1.978) (*P* = .640). This suggests that, although publication bias is present, the pooled effect remains stable.

#### 3.4.3. Comparison of the effects of HIIT and MICT on cardiorespiratory function in healthy elderly individuals

**Figure 5.**
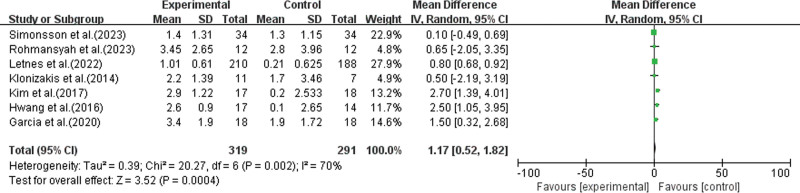
Comparative analysis of HIIT and MICT on cardiopulmonary function in healthy elderly). Direct comparison of 7 head to head studies (n = 610). HIIT significantly improves VO_2max_ compared to MICT [WMD = 1.17, 95% CI: 0.52–1.82, *P* < .01]. HIIT = high-intensity interval training, MICT = moderate-intensity continuous training, VO_2max_ = maximal oxygen uptake, WMD = weighted mean difference.

In this study, 7 studies simultaneously employed both HIIT and MICT as exercise interventions to evaluate the effects on cardiorespiratory function in 610 healthy elderly individuals.^[34,37,38,40,42,46,47]^ The meta-analysis results (as shown in Fig. 5) revealed a pooled effect size of: MD = 1.17, 95% CI: 0.52 to 1.82, *P* < .01, indicating statistically significant differences. This suggests that, compared to MICT, HIIT is more effective in promoting improvements in the cardiorespiratory function of healthy elderly individuals.

Furthermore, the heterogeneity test results showed *I*^2^ = 70%, *P *= .002 (<0.01), indicating significant heterogeneity among the study results. To assess the potential for publication bias, Egger test and Begg test were performed on the included studies. The results of Egger test showed *P* = .425 (*P* > .05), and the results of Begg test showed *P* = .548 (*P* > .05), indicating no publication bias and confirming the reliability of the results.

#### 3.4.4. Comparison of the effects of MICT and HIIT on cardiorespiratory function in healthy elderly individuals under different intervention parameters

According to the subgroup analysis results in Table 3, which examine the effects of exercise interventions under different parameters, the following findings were observed:

**Table 3:** Analysis of impact of MICT vs HIIT on heart and lung health of healthy elderly.

Subgroup			No. of trials/total no. (%)	Subjects (n)	MD (95% CI)	*P*-value	*P* for interaction
All studies			16/16 (100)	1024	1.33 [1.06 to 1.59]	<.00001	
Intervention cycle							
≤3 mo	MICT		4/12 (33)	73	0.46 [−0.55 to 1.47]	.38	.004^*^
	HIIT		7/11 (64)	121	2.56 [1.52 to 3.60]	<.00001	
>3 mo < 6 mo	MICT		3/12 (25)	64	2.53 [0.35 to 4.72]	.02	.64
	HIIT		2/11 (18)	30	3.42 [0.39 to 6.44]	.03	
≥6 mo	MICT		5/12 (42)	404	1.25 [0.67 to 1.82]	<.00001	.96
	HIIT		2/11 (18)	332	1.23 [0.62 to 1.83]	<.00001	
Intervention frequency							
2 times/wk	MICT		5/12 (42)	308	1.30 [0.12 to 2.48]	.3	.67
	HIIT		4/11 (36)	284	1.66 [0.43 to 2.90]	.008	
3 times/wk	MICT		5/12 (42)	201	1.34 [1.00 to 1.68]	<.00001	.79
	HIIT		5/11 (46)	165	1.44 [0.78 to 2.10]	<.00001	
4 times/wk	MICT		2/12 (17)	32	0.20 [−0.95 to 1.34]	.74	.007^*^
	HIIT		2/11 (18)	34	2.71 [1.29 to 4.12]	.0002	
Intervention duration							
Short time	MICT (≥20 min < 40 min)		3/12 (25)	60	1.27 [−0.75 to 3.28]	.22	.30
	HIIT (≤20 min)		6/11 (55)	104	2.54 [1.25 to 3.83]	.0001	
Subgroup			No. of trials/total no. (%)	Subjects (n)	MD (95% CI)	*P*-value	*P* for interaction
Medium time		MICT (≥40 min < 60 min)	7/12 (58)	429	1.08 [0.50 to 1.65]	.0003	.07
		HIIT (>20 min < 40 min)	2/11 (18)	29	2.69 [1.02 to 4.35]	.002	
Long time		MICT (≥60 min)	2/12 (17)	52	2.48 [0.15 to 4.82]	.04	.33
		HIIT (≥40 min)	3/11 (27)	350	1.28 [0.69 to 1.88]	<.00001	

95% CI = 95% confidence intervals, HIIT = high-intensive interval training, MD = mean difference, MICT = moderate-intensity continuous training.

* *P* < .05.

Intervention duration: HIIT exhibited a “short-duration, high-efficiency” characteristic. The best effect was achieved when the single session duration was between 20 and 39 minutes (WMD = 2.69, 95% CI: 1.02–4.35). In contrast, MICT achieved maximum benefit only when the session duration was ≥60 minutes (WMD = 2.48, 95% CI: 0.15–4.82).
Intervention duration (cycle): HIIT showed significant improvements in cardiorespiratory function in healthy elderly individuals within a short period (≤3 months) (MD = 2.56, 95% CI: 1.52–3.60). Furthermore, as the training period progressed, the best training effect was achieved during the mid-cycle period (>3 months and <6 months) (MD = 3.42, 95% CI: 0.39–6.44). In contrast, MICT did not show significant improvements in the short term (≤3 months). Its effects became significant only during the mid-cycle period (>3 months and <6 months), where the best results were observed (MD = 2.53, 95% CI: 0.35–4.72).
Intervention frequency: HIIT achieved the best effect with a higher frequency (4 times per week) (MD = 2.71, 95% CI: 1.29–4.12). For MICT, the best effect was seen with a moderate frequency (3 times per week) (MD = 1.34, 95% CI: 1.00–1.68). However, when MICT was conducted at a higher frequency (4 times per week), the training effect significantly declined (MD = 0.20, 95% CI: −0.95–1.34). This could be because high-frequency HIIT is more likely to disrupt homeostasis, inducing adaptive changes, while MICT at high frequencies with prolonged durations leads to reduced effectiveness due to fatigue accumulation.

In summary, the best training effect for HIIT was achieved with an intervention period of >3 months and <6 months, with 4 sessions per week and a single session duration of 21 to 39 minutes. For MICT, the best training effect was observed with an intervention period of >3 months and <6 months, with 3 sessions per week and a session duration of ≥60 minute.

### 3.5. Sensitivity analysis

Sensitivity analysis was performed on studies using MICT and HIIT as different exercise interventions, as well as studies that simultaneously used both HIIT and MICT as interventions. The results indicated that after sequentially removing each study, the overall effect size did not show significant changes, suggesting that the combined intervention results for MICT and HIIT, as well as the comparison of HIIT and MICT interventions, have a high level of robustness.

## 4. Discussion

This study provides a comprehensive and quantitative evaluation of the effects of HIIT and MICT on the cardiorespiratory function of healthy elderly individuals. The results indicate that both HIIT (MD = 1.62, 95% CI: 1.10–2.13, *P *< .00001) and MICT (MD = 1.22, 95% CI: 0.91–1.53, *P *< .00001) can significantly improve cardiorespiratory function in healthy elderly individuals. Compared to MICT, HIIT is more effective in improving cardiorespiratory function in healthy elderly individuals (MD = 1.17, 95% CI: 0.52–1.82). Subgroup analysis further identified the optimal intervention parameters for HIIT and MICT in improving cardiorespiratory function in healthy elderly individuals. The best intervention parameters for HIIT were: moderate duration (>3 months and <6 months), 4 sessions per week, with each session lasting 21 to 39 minutes; for MICT, the optimal intervention parameters were: moderate duration (>3 months and <6 months), 3 sessions per week, with each session lasting ≥60 minutes.

Currently, there are relatively few meta-analyses examining the effects of HIIT and MICT on the cardiorespiratory function of elderly individuals. Bouasiz et al meta-analysis suggests that both HIIT and MICT effectively improve cardiorespiratory health in elderly individuals,^[49]^ which aligns with the findings of this study. However, their analysis only included 3 RCTs, limiting the statistical power. In contrast, Cao et al meta-analysis focused on adolescents (ages 10–18), finding that both HIIT and MICT effectively improve cardiorespiratory fitness, with HIIT showing superior effects (WMD = 0.51, 95% CI = 0.33–0.69).^[25]^ In comparison, our study mainly included healthy elderly individuals, whose physiological function is generally weaker than that of adolescents. However, the results of this study show that with well-designed training plans tailored to individual physiological characteristics, healthy elderly individuals can safely and effectively improve their cardiorespiratory function through HIIT.

### 4.1. Potential mechanisms

#### 4.1.1. Mechanism analysis of MICT promoting the improvement of cardiopulmonary function in healthy elderly

In the results of this study, MICT significantly improved the cardiorespiratory function of healthy elderly individuals. Based on existing research findings, MICT can provide sustained stimulation to myocardial fibers through continuous aerobic exercise, promoting incremental regulation of left ventricular wall thickness and end diastolic volume, thereby increasing cardiac output.^[50]^ Long-term MICT training can reduce sarcoplasmic reticulum calcium permeability in myocardial cells and inhibit arrhythmogenic Ca^2+^ sparks, thereby improving cardiac contractile function and increasing stroke output.^[51]^ In addition, MICT can increase the proportion of type I muscle fibers (slow twitch muscle fibers) and the activity of succinate dehydrogenase enhance the β-oxidation ability of skeletal muscle to fatty acids, and promote the improvement of aerobic metabolism efficiency during exercise.^[44]^ MICT can also activate peroxisome proliferator-activated receptor gamma co activator 1-alpha through the adenosine 5’-monophosphate-activated protein kinase (AMPK) dependent pathway, promoting mitochondrial DNA replication and respiratory chain complex synthesis, and enhancing oxidative phosphorylation ability.^[52]^

The results of subgroup analysis showed that a single training period of 60 minutes and more than 12 weeks had the best effect on promoting the cardiorespiratory function of healthy elderly individuals. The potential mechanism is that the laminar shear force generated by MICT training for more than 60 minutes could maximize the activation of endothelial nitric oxide synthase, thereby improving the vasodilation function.^[53]^ Meanwhile, the 6-month MICT intervention upregulated skeletal muscle capillary density and fatty acid transporter protein expression, thereby optimizing oxygen utilization efficiency and ultimately enhancing cardiopulmonary function.^[54]^

#### 4.1.2. Mechanism analysis of HIIT promoting the improvement of cardiopulmonary function in healthy elderly

In the results of this study, HIIT can significantly improve the cardiopulmonary function of healthy elderly people. This is because HIIT can effectively activate mitochondrial biogenesis and inhibit inflammatory factors by alternating short-term high-intensity exercise with low-intensity recovery, thereby improving the cardiopulmonary function of the elderly.^[55]^ However, the training intensity and duration of HIIT need to be adjusted according to the specific situation of individuals to ensure the safety and effectiveness of the training process.^[56]^

Compared to MICT, HIIT has a better effect on improving cardiovascular and pulmonary function in healthy elderly people. Multiple experiments have explored the physiological mechanisms involved, among which Klonizakis et al study suggests that HIIT can achieve the same cardiorespiratory fitness improvement effect as MICT with shorter time and lower training volume.^[46]^ This is partly because HIIT training can significantly increase blood flow mediated vasodilation, reduce arterial stiffness, promote the release of nitric oxide, improve vascular elasticity,^[57]^ and promote the regeneration of skeletal muscle capillaries by upregulating the expression of vascular endothelial growth factor, shorten the diffusion distance of oxygen from blood to mitochondria, thereby improving vascular endothelial function.^[58]^ The RCTs by Wisloff et al^[59]^ showed that HIIT with exercise intensity of 90% VO_2max_ was more effective than MICT with exercise intensity of 70% VO_2max_ in improving cardiopulmonary function. This study suggests that high-intensity exercise, compared to moderate to low-intensity exercise, can induce an increase in cardiac output, thereby promoting the efficiency of oxygen transport per unit time and resulting in better improvement of cardiopulmonary function. In addition, in Maclnnis et al study, participants were trained with HIIT and MICT on both legs, and the results showed that HIIT promoted a more significant increase in mitochondrial content compared to MICT.^[50]^ This is because HIIT can transiently activate the AMPK/PGC-1α (peroxisome proliferator-activated receptor gamma coactivator 1-alpha) signaling pathway, thereby increasing the rate of mitochondrial biosynthesis in skeletal muscle.

Subgroup analysis revealed that HIIT elicits rapid and efficient improvements in cardiorespiratory function among healthy older adults. This is because 30 minutes HIIT training can upregulate mitochondrial fission genes (drp1/fis1), promote the renewal of damaged mitochondria, and enhance the expression of fusion genes (mfn2/opa1) to maintain network integrity.^[60]^ At the same time, more than 40 minutes will induce excessive division and reduce function. At the same time, HIIT with high frequency can promote the phosphorylation level of AMPK to rise steadily, avoiding the signal attenuation in the intermittent period.^[61]^

### 4.2. Strengths and limitations

This study is the 1st to focus on the effects of MICT and HIIT on the cardiorespiratory health of healthy elderly individuals. The findings confirm that, with consideration of individual physiological characteristics and scientifically designed training plans, HIIT can be safely and effectively used to improve the cardiorespiratory function of healthy elderly individuals, just as it does for adolescents and adults. Furthermore, this study systematically quantified the optimal intervention parameters for MICT and HIIT, providing important practical evidence for clinical practice and exercise prescription development.

However, despite strictly adhering to the PRISMA guidelines for evaluation, there are still some limitations in this study:

1. There were differences in the specific implementation protocols of HIIT and MICT in the included studies, leading to a high heterogeneity index when comparing the effect sizes of HIIT and MICT. Future research should aim to standardize training protocols to reduce heterogeneity.
2. Although sensitivity analysis verified the reliability of the results, 2 of the included studies had a high risk of bias, and only 4 studies had a low risk of bias, which may reduce the methodological quality.
3. Publication bias was detected in the HIIT group via Egger test, and although the trim-and-fill method confirmed the robustness of the results, the findings should be interpreted with caution.
4. The studies included in this analysis primarily focused on healthy elderly individuals, excluding those with chronic diseases or limited physical capacity, which may limit the generalizability of the results to a broader elderly population.
5. Although the study provided insights into the physiological mechanisms of MICT and HIIT on improving cardiorespiratory health in the elderly, direct evidence, such as biomarkers, is still lacking, and further clinical research is needed to validate these findings.

Future research should optimize study designs to address the limitations of the current study: standardize the intervention parameters of HIIT and MICT to reduce heterogeneity and enhance the credibility of the results; continue to increase the sample size and include elderly individuals with chronic diseases to improve the external validity of the findings; further explore the effects of MICT and HIIT on other aspects of elderly health to increase the practical impact of research outcomes in improving elderly health.

## 5. Conclusion

This meta-analysis systematically evaluated the effects of HIIT and MICT on cardiorespiratory function in elderly populations. The results confirm that both training modalities are effective in improving key indicators of cardiorespiratory function in older adults; notably, HIIT exhibits superior improvement efficiency in short-term interventions. Additionally, this study identified the optimal training parameters for both modalities, providing a quantitative basis for personalized exercise prescription tailored to the diverse health needs of older adults.

To translate these findings into actionable recommendations for clinicians, community health workers, and exercise instructors, targeted protocols are proposed based on older adults’ health status and fitness level. For those with good baseline fitness, HIIT is recommended for efficient, short-term improvement: 4 sessions per week, 21 to 39 minutes per session, and a 3 to 6 month program, with prior health screening and heart rate monitoring for safety. For those with lower exercise tolerance, MICT is more suitable for stable, long-term adaptation: 3 sessions per week, ≥60 minutes per session, a ≥6 month program, and precautions like avoiding extreme weather and choosing safe venues. For those with severe chronic conditions, medical supervised low-intensity MICT (e.g., slow walking, chair-based aerobics) is advised: starting with 20 to 30 minutes per session, gradually increasing to 45 minutes, and requiring real-time professional supervision to monitor vital signs.

Beyond specific training protocols, several principles are critical to maximizing benefits and ensuring safety for older adults. Individualization is key: the choice between HIIT and MICT should integrate not only health status but also personal preferences (e.g., preference for short intense workouts vs long steady sessions) and environmental conditions (e.g., access to equipment for HIIT vs outdoor spaces for MICT): for example, those in high-rises without elevators may prioritize indoor cycling for MICT to avoid stair risks. Progressive overload is also essential: after 4 weeks of HIIT adaptation, gradually increase high-intensity duration or reduce recovery time; for MICT, add 5 to 10 minutes per session every 2 weeks or slightly raise intensity if tolerated. To address low adherence, incorporating social elements (e.g., community group sessions) or goal-setting (e.g., “12 MICT sessions monthly for a reward”) and regular follow-ups (e.g., monthly health worker check-ins) can help overcome barriers like joint pain or time constraints.

While this study provides evidence-based exercise parameters, limitations remain to guide future research. First, included studies focused primarily on healthy or mildly impaired older adults, with insufficient data on those with severe chronic diseases (e.g., end-stage renal disease): expanding the sample scope is needed to validate HIIT/MICT safety and efficacy in more diverse groups. Second, long-term follow-up data (≥1 year) is lacking to assess the sustainability of cardiorespiratory improvements; longitudinal studies should explore if maintenance training (e.g., reducing HIIT to 2 sessions weekly after 6 months) preserves benefits. Finally, combining HIIT/MICT with resistance training (e.g., bodyweight exercises) may yield synergistic effects on both cardiorespiratory and muscular function, a direction that warrants further investigation to enhance the comprehensiveness of exercise prescriptions for older adults.

## Author contributions

**Formal analysis:** Rui Chu, Mingming Li.

**Funding acquisition:** Mingming Li.

**Investigation:** Mingming LI, Caiwei Zhu.

**Resources:** Rui Chu.

**Software:** Rui Chu.

**Supervision:** Caiwei Zhu, Shouzhi Wu.

**Writing – original draft:** Rui Chu.

**Writing – review & editing:** Rui Chu, Mingming Li, Yinuo Du.

## References

[1] Sui X LaMonte MJ Blair SN. Cardiorespiratory fitness as a predictor of nonfatal cardiovascular events in asymptomatic women and men. Am J Epidemiol. 2007;165:1413–23. 17406007 10.1093/aje/kwm031 PMC2685148

[2] Ross R Blair SN Arena R. Importance of assessing cardiorespiratory fitness in clinical practice: a case for fitness as a clinical vital sign: a scientific statement from the American Heart Association. Circulation. 2016;134:e653–99. 27881567 10.1161/CIR.0000000000000461

[3] Sui X LaMonte MJ Laditka JN. Cardiorespiratory fitness and adiposity as mortality predictors in older adults. JAMA. 2007;298:2507–16. 18056904 10.1001/jama.298.21.2507 PMC2692959

[4] Blair SN Kohl HW 3rd Paffenbarger RS Jr Clark DG Cooper KH Gibbons LW. Physical fitness and all-cause mortality. A prospective study of healthy men and women. JAMA. 1989;262:2395–401. 2795824 10.1001/jama.262.17.2395

[5] Gupta S Rohatgi A Ayers CR. Cardiorespiratory fitness and classification of risk of cardiovascular disease mortality. Circulation. 2011;123:1377–83. 21422392 10.1161/CIRCULATIONAHA.110.003236 PMC3926656

[6] Cote CG Pinto-Plata V Kasprzyk K Dordelly LJ Celli BR. The 6-min walk distance, peak oxygen uptake, and mortality in COPD. Chest. 2007;132:1778–85. 17925409 10.1378/chest.07-2050

[7] Dong C Rundek T Wright CB Anwar Z Elkind MS Sacco RL. Ideal cardiovascular health predicts lower risks of myocardial infarction, stroke, and vascular death across whites, blacks, and hispanics: the northern Manhattan study. Circulation. 2012;125:2975–84. 22619283 10.1161/CIRCULATIONAHA.111.081083 PMC3396556

[8] Folsom AR Yatsuya H Nettleton JA Lutsey PL Cushman M Rosamond WD. Community prevalence of ideal cardiovascular health, by the American Heart Association definition, and relationship with cardiovascular disease incidence. J Am Coll Cardiol. 2011;57:1690–6. 21492767 10.1016/j.jacc.2010.11.041 PMC3093047

[9] Jackson AS Sui X Hébert JR Church TS Blair SN. Role of lifestyle and aging on the longitudinal change in cardiorespiratory fitness. Arch Intern Med. 2009;169:1781–7. 19858436 10.1001/archinternmed.2009.312 PMC3379873

[10] Ito S. High-intensity interval training for health benefits and care of cardiac diseases - The key to an efficient exercise protocol. World J Cardiol. 2019;11:171–88. 31565193 10.4330/wjc.v11.i7.171 PMC6763680

[11] Hannan AL Hing W Simas V. High-intensity interval training versus moderate-intensity continuous training within cardiac rehabilitation: a systematic review and meta-analysis. Open Access J Sports Med. 2018;9:1–17. 29416382 10.2147/OAJSM.S150596 PMC5790162

[12] Buchheit M Laursen PB. High-intensity interval training, solutions to the programming puzzle. Part II: anaerobic energy, neuromuscular load and practical applications. Sports Med. 2013;43:927–54. 23832851 10.1007/s40279-013-0066-5

[13] Hickson RC Bomze HA Holloszy JO. Linear increase in aerobic power induced by a strenuous program of endurance exercise. J Appl Physiol Respir Environ Exerc Physiol. 1977;42:372–6. 838658 10.1152/jappl.1977.42.3.372

[14] Talanian JL Galloway SD Heigenhauser GJ Bonen A Spriet LL. Two weeks of high-intensity aerobic interval training increases the capacity for fat oxidation during exercise in women. J Appl Physiol (1985). 2007;102:1439–47. 17170203 10.1152/japplphysiol.01098.2006

[15] de Queiros VS Rolnick N Sabag A. Effect of high-intensity interval exercise versus continuous low-intensity aerobic exercise with blood flow restriction on psychophysiological responses: a randomized crossover study. J Sports Sci Med. 2024;23:114–25. 38455431 10.52082/jssm.2024.114 PMC10915608

[16] Bonet JB Magalhães J Viscor G Pagès T Javierre C Torrella JR. High-intensity interval versus moderate-intensity continuous half-marathon training programme for middle-aged women. Eur J Appl Physiol. 2020;120:1083–96. 32193662 10.1007/s00421-020-04347-z

[17] Grace F Herbert P Elliott AD Richards J Beaumont A Sculthorpe NF. High intensity interval training (HIIT) improves resting blood pressure, metabolic (MET) capacity and heart rate reserve without compromising cardiac function in sedentary aging men. Exp Gerontol. 2018;109:75–81. 28511954 10.1016/j.exger.2017.05.010

[18] Arild A Vangberg T Nikkels H. Five years of exercise intervention at different intensities and development of white matter hyperintensities in community dwelling older adults, a Generation 100 sub-study. Aging (Albany NY). 2022;14:596–622. 35042832 10.18632/aging.203843 PMC8833118

[19] Matsuo T Saotome K Seino S. Effects of a low-volume aerobic-type interval exercise on VO_2max_ and cardiac mass. Med Sci Sports Exerc. 2014;46:42–50. 23846165 10.1249/MSS.0b013e3182a38da8

[20] Tsai HH Lin CP Lin YH Hsu CC Wang JS. High-intensity Interval training enhances mobilization/functionality of endothelial progenitor cells and depressed shedding of vascular endothelial cells undergoing hypoxia. Eur J Appl Physiol. 2016;116:2375–88. 27761657 10.1007/s00421-016-3490-z

[21] Cocks M Shaw CS Shepherd SO. Sprint interval and endurance training are equally effective in increasing muscle microvascular density and eNOS content in sedentary males. J Physiol. 2013;591:641–56. 22946099 10.1113/jphysiol.2012.239566 PMC3577551

[22] Dunham C Harms CA. Effects of high-intensity interval training on pulmonary function. Eur J Appl Physiol. 2012;112:3061–8. 22194005 10.1007/s00421-011-2285-5

[23] Gillen JB Martin BJ MacInnis MJ Skelly LE Tarnopolsky MA Gibala MJ. Twelve weeks of sprint interval training improves indices of cardiometabolic health similar to traditional endurance training despite a five-fold lower exercise volume and time commitment. PLoS One. 2016;11:e0154075. 27115137 10.1371/journal.pone.0154075 PMC4846072

[24] Poon ET Wongpipit W Ho RS Wong SH. Interval training versus moderate-intensity continuous training for cardiorespiratory fitness improvements in middle-aged and older adults: a systematic review and meta-analysis. J Sports Sci. 2021;39:1996–2005. 33825615 10.1080/02640414.2021.1912453

[25] Cao M Quan M Zhuang J. Effect of high-intensity interval training versus moderate-intensity continuous training on cardiorespiratory fitness in children and adolescents: a meta-analysis. Int J Environ Res Public Health. 2019;16:1533. 31052205 10.3390/ijerph16091533 PMC6539300

[26] García-Hermoso A Cerrillo-Urbina AJ Herrera-Valenzuela T Cristi-Montero C Saavedra JM Martínez-Vizcaíno V. Is high-intensity interval training more effective on improving cardiometabolic risk and aerobic capacity than other forms of exercise in overweight and obese youth? A meta-analysis. Obes Rev. 2016;17:531–40. 26948135 10.1111/obr.12395

[27] Liberati A Altman DG Tetzlaff J. The PRISMA statement for reporting systematic reviews and meta-analyses of studies that evaluate healthcare interventions: explanation and elaboration. BMJ. 2009;339:b2700. 19622552 10.1136/bmj.b2700 PMC2714672

[28] Weston M Taylor KL Batterham AM Hopkins WG. Effects of low-volume high-intensity interval training (HIT) on fitness in adults: a meta-analysis of controlled and non-controlled trials. Sports Med. 2014;44:1005–17. 24743927 10.1007/s40279-014-0180-z PMC4072920

[29] Norton K Norton L Sadgrove D. Position statement on physical activity and exercise intensity terminology. J Sci Med Sport. 2010;13:496–502. 20005170 10.1016/j.jsams.2009.09.008

[30] Buchheit M Laursen PB. High-intensity interval training, solutions to the programming puzzle: Part I: cardiopulmonary emphasis. Sports Med. 2013;43:313–38. 23539308 10.1007/s40279-013-0029-x

[31] Hedges LV Olkin I. Statistical methods for meta-analysis. Academic press; 2014.

[32] Egger M Smith GD Schneider M Minder C. Bias in meta-analysis detected by a simple, graphical test. BMJ. 1997;315:629–34. 9310563 10.1136/bmj.315.7109.629 PMC2127453

[33] Uusitalo AL Laitinen T Väisänen SB Länsimies E Rauramaa R. Effects of endurance training on heart rate and blood pressure variability. Clin Physiol Funct Imaging. 2002;22:173–9. 12076342 10.1046/j.1475-097x.2002.00414.x

[34] Simonsson E Sandström SL Hedlund M. Effects of controlled supramaximal high-intensity interval training on cardiorespiratory fitness and global cognitive function in older adults: the umeå HIT study – A randomized controlled trial. J Gerontol A Biol Sci Med Sci. 2023;78(9):1581–1590. 36972981 10.1093/gerona/glad070 PMC10460559

[35] Haynes A Naylor LH Carter HH. Land-walking vs. water-walking interventions in older adults: Effects on aerobic fitness. J Sport Health Sci. 2020;9:274–82. 32444152 10.1016/j.jshs.2019.11.005 PMC7242220

[36] Sian TS Inns TB Gates A. Equipment-free, unsupervised high intensity interval training elicits significant improvements in the physiological resilience of older adults. BMC Geriatr. 2022;22:529. 35761262 10.1186/s12877-022-03208-y PMC9238013

[37] Letnes JM Berglund I Johnson KE. Effect of 5 years of exercise training on the cardiovascular risk profile of older adults: the Generation 100 randomized trial. Eur Heart J. 2022;43:2065–75. 34746955 10.1093/eurheartj/ehab721 PMC9156390

[38] Hwang CL Yoo JK Kim HK. Novel all-extremity high-intensity interval training improves aerobic fitness, cardiac function and insulin resistance in healthy older adults. Exp Gerontol. 2016;82:112–9. 27346646 10.1016/j.exger.2016.06.009 PMC4975154

[39] Frost NJ Weinborn M Gignac GE. A randomized controlled trial of high-intensity exercise and executive functioning in cognitively normal older adults. Am J Geriatr Psychiatry. 2021;29:129–40. 32732104 10.1016/j.jagp.2020.06.015

[40] Ballesta-García I Martínez-González-Moro I Ramos-Campo DJ Carrasco-Poyatos M. High-intensity interval circuit training versus moderate-intensity continuous training on cardiorespiratory fitness in middle-aged and older women: a randomized controlled trial. Int J Environ Res Public Health. 2020;17:1805. 32164314 10.3390/ijerph17051805 PMC7084372

[41] Fosstveit SH Berntsen S Feron J. HIIT at home: enhancing cardiorespiratory fitness in older adults – a randomized controlled trial. Scand J Med Sci Sports. 2024;34:e14694. 38982665 10.1111/sms.14694

[42] Rohmansyah NA Ka Praja R Phanpheng Y Hiruntrakul A. High-intensity interval training versus moderate-intensity continuous training for improving physical health in elderly women. Inquiry. 2023;60:469580231172870. 37158072 10.1177/00469580231172870 PMC10184247

[43] Brown BM Frost N Rainey-Smith SR. High-intensity exercise and cognitive function in cognitively normal older adults: a pilot randomised clinical trial. Alzheimers Res Ther. 2021;13:33. 33522961 10.1186/s13195-021-00774-y PMC7849126

[44] Guadagni V Drogos LL Tyndall AV. Aerobic exercise improves cognition and cerebrovascular regulation in older adults. Neurology. 2020;94:e2245–57. 32404355 10.1212/WNL.0000000000009478 PMC7357295

[45] Herrod PJJ Blackwell JEM Boereboom CL. The time course of physiological adaptations to high-intensity interval training in older adults. Aging Med (Milton). 2020;3:245–51. 33392430 10.1002/agm2.12127 PMC7771560

[46] Klonizakis M Moss J Gilbert S Broom D Foster J Tew GA. Low-volume high-intensity interval training rapidly improves cardiopulmonary function in postmenopausal women. Menopause. 2014;21:1099–105. 24552980 10.1097/GME.0000000000000208

[47] Kim HK Hwang CL Yoo JK. All-extremity exercise training improves arterial stiffness in older adults. Med Sci Sports Exerc. 2017;49:1404–11. 28166118 10.1249/MSS.0000000000001229 PMC5474160

[48] Herbert P Hayes LD Beaumont AJ Grace FM Sculthorpe NF. Six weeks of high intensity interval training (HIIT) facilitates a four year preservation of aerobic capacity in sedentary older males: a reunion study. Exp Gerontol. 2021;150:111373. 33895265 10.1016/j.exger.2021.111373

[49] Bouaziz W Malgoyre A Schmitt E Lang PO Vogel T Kanagaratnam L. Effect of high-intensity interval training and continuous endurance training on peak oxygen uptake among seniors aged 65 or older: a meta-analysis of randomized controlled trials. Int J Clin Pract. 2020;74:e13490. 32083390 10.1111/ijcp.13490

[50] MacInnis MJ Zacharewicz E Martin BJ. Superior mitochondrial adaptations in human skeletal muscle after interval compared to continuous single-leg cycling matched for total work. J Physiol. 2017;595:2955–68. 27396440 10.1113/JP272570 PMC5407978

[51] Villelabeitia-Jaureguizar K Vicente-Campos D Senen AB Jiménez VH Garrido-Lestache MEB Chicharro JL. Effects of high-intensity interval versus continuous exercise training on post-exercise heart rate recovery in coronary heart-disease patients. Int J Cardiol. 2017;244:17–23. 28648356 10.1016/j.ijcard.2017.06.067

[52] Bode D Rolim NPL Guthof T. Effects of different exercise modalities on cardiac dysfunction in heart failure with preserved ejection fraction. ESC Heart Fail. 2021;8:1806–18. 33768692 10.1002/ehf2.13308 PMC8120378

[53] Pirani H Bakhtiari A Amiri B Salehi OR. Beneficial mitochondrial biogenesis in gastrocnemius muscle promoted by high-intensity interval training in elderly female rats. Cell J. 2023;25:11–6. 36680479 10.22074/CELLJ.2022.557565.1078 PMC9868433

[54] Shi W Chen J He Y. The effects of high-intensity interval training and moderate-intensity continuous training on visceral fat and carotid hemodynamics parameters in obese adults. J Exerc Sci Fit. 2022;20:355–65. 36186829 10.1016/j.jesf.2022.09.001 PMC9486563

[55] Huenchullán SM. Exercise intensity-dependent metabolic benefits in muscle, adipose tissue, and liver of candidates to undergo bariatric surgery (Doctoral dissertation, Faculty of Medicine, Universidad Austral de Chile, Valdivia, Chile).

[56] Sert H Eren MG Gurcay B Koc F. The effectiveness of a high-intensity interval exercise on cardiometabolic health and quality of life in older adults: a systematic review and meta-analysis. BMC Sports Sci Med Rehabil. 2025;17:128. 40413509 10.1186/s13102-025-01176-5 PMC12102952

[57] Ramos JS Dalleck LC Tjonna AE Beetham KS Coombes JS. The impact of high-intensity interval training versus moderate-intensity continuous training on vascular function: a systematic review and meta-analysis. Sports Med. 2015;45:679–92. 25771785 10.1007/s40279-015-0321-z

[58] Torma F Gombos Z Jokai M Takeda M Mimura T Radak Z. High intensity interval training and molecular adaptive response of skeletal muscle. Sports Med Health Sci. 2019;1:24–32. 35782463 10.1016/j.smhs.2019.08.003 PMC9219277

[59] Wisløff U Støylen A Loennechen JP. Superior cardiovascular effect of aerobic interval training versus moderate continuous training in heart failure patients: a randomized study. Circulation. 2007;115:3086–94. 17548726 10.1161/CIRCULATIONAHA.106.675041

[60] Jahangiri M Shahrbanian S Gharakhanlou R. High intensity interval training alters gene expression linked to mitochondrial biogenesis and dynamics in high fat diet fed rats. Sci Rep. 2025;15:5442. 39952980 10.1038/s41598-025-86767-5 PMC11828894

[61] Mahatme S K V Kumar N Rao V Kovela RK Sinha MK. Impact of high-intensity interval training on cardio-metabolic health outcomes and mitochondrial function in older adults: a review. Med Pharm Rep. 2022;95:115–30. 35721039 10.15386/mpr-2201 PMC9176307

